# Straw and phosphorus applications promote maize (*Zea mays* L.) growth in saline soil through changing soil carbon and phosphorus fractions

**DOI:** 10.3389/fpls.2024.1336300

**Published:** 2024-01-19

**Authors:** Zhenxin Guo, Wenhua Ye, Hui Wang, Wei He, Yanling Tian, Guoqing Hu, Yanhong Lou, Hong Pan, Quangang Yang, Yuping Zhuge

**Affiliations:** ^1^ National Engineering Research Center for Efficient Utilization of Soil and Fertilizer Resources, College of Resources and Environment, Shandong Agricultural University, Taian, China; ^2^ Observation and Research Station of Land Use Security in the Yellow River Delta, Ministry of Natural Resources (NMR), Shandong Provincial Territorial Spatial Ecological Restoration Center, Jinan, China

**Keywords:** straw return, P addition, antioxidant enzyme, particulate organic carbon, soil P availability, saline soil

## Abstract

**Introduction:**

Straw return has been widely recognized as an important carbon (C) enhancement measure in agroecosystems, but the C-phosphorus (P) interactions and their effects on plants in saline soils are still unclear.

**Methods:**

In this study, we investigated the effects of straw return and three P application levels, no P fertilizer (Non-P), a conventional application rate of P fertilizer (CP), and a high application rate of P fertilizer (HP), on maize growth and soil C and P fractions through a pot experiment.

**Results and discussion:**

The results revealed that the dry matter weight of maize plant was no difference between the two straw return levels and was 15.36% higher under HP treatments than under Non-P treatments. Plant nutrient accumulations were enhanced by straw addition and increased with increasing P application rate. Straw application reduced the activities of peroxidase (POD), superoxide dismutase (SOD), catalase, and the content of malondialdehyde (MDA) in maize plants by 31.69%, 38.99%, 45.96% and 27.04%, respectively. P application decreased SOD, POD activities and MDA content in the absence of straw. The contents of easily oxidized organic carbon (EOC), particulate organic carbon (POC) and the ratio of POC/SOC in straw-added soils were 10.23%, 17.00% and 7.27% higher, respectively, than those in straw-absent soils. Compared with Non-P treatments, HP treatments led to an increase of 12.05%, 23.04% in EOC, POC contents respectively, while a decrease of 18.12% in the contribution of MAOC to the SOC pool. Straw return improved the P status of the saline soil by increasing soil available P (14.80%), organic P (35.91%) and Ca_2_-P contents (4.68%). The structural equation model showed that straw and P applications could promote maize growth (indicated by dry matter weight, P accumulation, antioxidant enzyme activity and MDA content) through improving soil C and P availabilities.

**Conclusion:**

This study provides evidence that straw return together with adequate P supply in saline soil can promote crop nutrient accumulation, attenuate the oxidation damage on crop growth, and be beneficial for SOC turnover and soil P activation.

## 1 Introduction

Saline soils are widely distributed in the world, with a total area of approximately 833 million hectares, accounting for 8.7% of the world’s land area (FAO, 2021). The fertility and productivity of saline soil are usually low, which affects regional economic and social development. It is estimated that on average, 3.47 t of soil organic carbon (SOC) is lost per hectare of soil due to salinization (Setia et al., 2013). On one hand, salt may reduce the productivity of crops, thereby reducing plant residue contributions to soil organic matter (SOM) (Li et al., 2006); on the other hand, the saline soil usually has a high sodium ion content, which leads to dispersion of soil particles and is not conducive to the formation of soil aggregates, thus weakening the physical protection of SOC and aggravating its loss. The application of organic materials, such as crop straw, manure, or compost, are widely applied as management measures to increase soil carbon (C) and reduce salinization (Ouni et al., 2013; Xie et al., 2021; Zhao et al., 2022).

Straw return is considered a reliable measure to improve the quality of saline soil (Xie et al., 2017), which can significantly ameliorate the physical, chemical and biological characteristics of the soil and promote crop growth in a relatively short term (Xiu et al., 2019; Bu et al., 2020). After entering the soil, the straw will be fragmented, decomposed and participate in the formation of different C fractions under the combined effects of biological and abiotic factors. Among the SOC fractions, particulate organic carbon (POC), which is susceptive to environmental change and represents unstable C components, can accumulate rapidly in the soil and can be used as an early indicator of the change in SOC (Pikul et al., 2007; Qu et al., 2021), and mineral-associated organic carbon (MAOC), which can exist in soil for a long time, is considered to be an important component maintaining the stability of the C pool and contributing to global climate change mitigation (Cai et al., 2016). Previous studies generally found that straw return could increase the content of unstable C components such as POC and easily oxidizable organic carbon (EOC) (Wu et al., 2021) and that of MAOC (He et al., 2022) in saline soils. However, for the proportions of SOC fraction, the straw return might increase (Li et al., 2019; Wang et al., 2020), be unaffected, or decrease (He et al., 2022) the contribution of POC to SOC.

Nutrient availability may be an important factor affecting soil C fractions allocation. An abundant supply of phosphorus (P) and nitrogen (N) has been shown to be necessary for the growth of soil microorganisms and to increase SOC sequestration (Kirkby et al., 2014). Some studies found that POC and MAOC contents increased with increasing N addition (Zhang et al., 2022a). The meta-analysis also showed that the C and N contents of the substrate could strongly affect the POC and MAOC contents (Zhang et al., 2022b). However, it has not been reported whether the composition of SOC fractions is affected by P availability under the condition of straw return in saline soil.

Nutrient availability is also an important factor affecting crop productivity. Saline soil has a low organic matter content and weak capacity for nutrient retention and supply and generally cannot provide enough nutrients for crops (Lakhdar et al., 2008). The saline soil of the Yellow River Delta is developed from the alluvial parent material of the Yellow River. Due to the high content of calcium carbonate in the soil, the fixation of soil water-soluble P (i.e., soil available P is transformed into insoluble P components such as Ca-P) is quite strong in this region (Guo et al., 2018), thus causing P deficiency in crops and making them more vulnerable to salt stress. A recent study by Cao et al. (2021) suggests that straw return combined with P fertilizer application can increase the phosphorus activation coefficient (i.e., the proportion of soil available P to total P) and improve the soil available P content in coastal saline soil. However, some studies do not support a clear effect of straw return on soil P availability (Khan et al., 2019). Furthermore, Li et al. (2015) found that straw return (20 years) showed a significant increase in subtropical paddy soil P accumulation (13% ~ 20%) but had no significant influence on inorganic P fractions. At present, it is still unclear how straw return affects P fraction distribution in saline soil and whether it can alleviate salt stress on crop growth.

Saline soils in the Yellow River Delta account for more than 70% of its terrestrial area (Li et al., 2020) and pose a great threat to soil fertility and crop production. In recent years, the comprehensive development and utilization of saline soils in the Yellow River Delta has become a national strategy in China. In this study, regarding the typical saline soil in the Yellow River Delta as the research object, the effects of straw return combined with P fertilizer on maize growth, soil C fractions and soil P fractions were investigated. The objectives of this study were (1) to clarify how straw return and P application affect maize growth and resistance to salt stress, (2) to analyze the effects of straw return and P application on SOC fractions, and (3) to explore whether P fraction allocation in saline soil varies with straw return level. We hypothesized that (1) the combination of straw return and P fertilizer application could promote maize growth and enhance the activity of plant antioxidants; (2) straw return would increase the proportion of soil readily available organic C (POC and EOC) and decrease that of MAOC, while P application would weaken this effect because the increase in P availability promotes microbial proliferation and increases the uptake of readily available organic C by microorganisms (Damon et al., 2014; Cao et al., 2021); and (3) straw returning could improve the P availability in saline soil, as indicated in most other soils (Damon et al., 2014).

## 2 Materials and methods

### 2.1 Soil and straw used

The saline soil used in this study was collected from the 0~20 cm soil layers of medium-salinity soil at the Wudi experimental station of Shandong Agricultural University (demonstration center of the “Bohai Granary” project), Binzhou, Shandong Province, China (37°54′40″-37°57′38″ N, 117°54′21″-117°56′40″ W) in September 2019. The soil has a silt texture comprising 7.1% sand (> 0.05 mm), 80.9% silt (0.002~0.05 mm) and 12.0% clay (< 0.002 mm) and was classified as Fluvic Cambisols (FAO soil classification). The basic physical and chemical properties of the study soil were as follows: pH 8.55, SOM 9.16 g kg^-1^, total N 0.63 g kg^-1^, available P 26.56 mg kg^-1^, available potassium (K) 232.67 mg kg^-1^ and electrical conductivity at a soil:water ratio of 1:5 (EC_1:5_) 1033.58 μs/cm. After collection, the soil was air-dried and finely ground to remove vegetation and stones and then passed through a sieve (2 mm). The wheat straw used in the pot experiment was oven-dried and ground through a 2 mm sieve. The straw contained 0.88% N, 0.14% P and 1.16% K.

### 2.2 Experimental design

The maize pot experiment was conducted at the experimental station of Shandong Agriculture University (36°17′ N, 117°15′ E). Six treatments with four replicates were set up: control (CK, without straw or P application), conventional P application (CP), high P application (HP), straw return (S), straw return with conventional P application (SCP), straw return with high P application (SHP). Briefly, one maize (*Zea mays L*., Ziyu 2) seedling was planted in a plastic basin (upper inner diameter 32 cm, lower inner diameter 26 cm, height 29 cm) filled with 15 kg of air-dried soil. The specific amounts of chemical fertilizer and straw are shown in **Table 1**. N fertilizer had a basal to the topdressing ratio of 1:1, and P and K fertilizers were all used as basal fertilizers. Topdressing was carried out at the big flare stage of maize. Fertilizers are urea (N 46%), diammonium phosphate (N 15%, P_2_O_5_ 42%) and potassium sulfate (K_2_O 50%). Seeds were sown on July 11th, 2020 and emerged on July 18th, 2020.

**Table 1:** Fertilizer and straw application rates in the pot experiment (kg hm^-2^).

Treatments	Wheat straw	N		P_2_O_5_	K_2_O
		Basal	Top dressing		
CK	0	112.5	112.5	0	75
CP	0	112.5	112.5	150	75
HP	0	112.5	112.5	300	75
S	6200	112.5	112.5	0	75
SCP	6200	112.5	112.5	150	75
SHP	6200	112.5	112.5	300	75

### 2.3 Nutrient accumulation

At the mature stage of maize growth, the maize plant was destructively sampled, washed with distilled water, and separated into three parts: root, straw (including stem, leaf and corn cob) and grain. The root, straw and grain were oven-dried at 75°C, weighed, and then milled. The total N, P and K contents in each part were determined by the Kjeldahl method, vanadium-molybdenum yellow colorimetric method and flame atomic absorption spectrophotometry method, respectively (Bao, 2000).

### 2.4 Antioxidant enzyme activities and MDA content

Fresh leaves (the ear leaf) of maize were taken at the tasseling stage for the determination of leaf antioxidant enzyme activities and malondialdehyde (MDA) content. Specifically, 0.2 g of fresh leaves were placed in a precooled mortar, and 1.8 mL phosphate buffer (pH = 7.8) was added to the ice bath to grind the leaves into a homogenate. The grinding solution was transferred into a 2 mL centrifuge tube and centrifuged at 12000 r·min^-1^ for 10 min at 4°C. The supernatant, which was the crude enzyme extract, was stored at -20°C for further use for the determination of peroxidase (POD) and superoxide dismutase (SOD) activities. Alternatively, the phosphate buffer used above was replaced with saline extract to obtain the enzyme extract for the determination of catalase (CAT) activity. The extract was replaced with the extract from the MDA kit, and the obtained enzyme extract was used for the determination of MDA content.

Then, the enzyme extracts were further processed for POD, SOD, CAT and MDA measurements using A084-3-1, T001-1, A007-1-1 and A003-3-1 kits (Nanjing Jiancheng Bioengineering Institute), respectively. Finally, the absorbance values were measured at 420 nm, 550 nm, 405 nm and 532 nm using a plate reader (Synergy HTX, BioTek, USA).

### 2.5 Determination of soil C and P fractions

After the maize plant was harvested, soil samples were collected, air-dried, and ground through 10 mesh, 20 mesh and 100 mesh sieves as needed to determine the contents of C fractions (including POC, MAOC and EOC) and P fractions (including soil organic P, inorganic P and available P). The soil EOC content was determined by the 0.02 mol·L^-1^ KMnO_4_ oxidation method (Weil et al., 2003). Briefly, 5 g air-dried soil was mixed with 20 mL 0.02 mol·L^-1^ KMnO_4_ solution and shook at 200 pm for 2 min, then centrifuged at 3000 rpm for 5 min. The supernatant was transferred and diluted, and the absorbance value was determined at 550 nm. Soil POC and MAOC content was measured by wet sieving-K_2_Cr_2_O_7_ oxidation method: 20 g air-dried soil which passed through 2 mm sieve was dispersed in 100 mL sodium hexametaphosphate (5%) for 18 h, and then sieved through a 53 μm sieve, the particles remaining on the sieve is POC fraction, and the particles passed through the 53 μm sieve is MAOC fraction. The POC fraction was oven-dried at 65°C for 48 h and then determined by the K_2_Cr_2_O_7_ oxidation method. MAOC was calculated by subtracting the POC content from the SOC content (Adams et al., 2015).

Soil available P (AP) was extracted with 0.5 mol L^-1^ NaHCO_3_ and determined by the molybdenum antimony colorimetric method (Bao, 2000). Inorganic P fractions were classified by the grading method for calcareous soils (Gu and Jiang, 1990). Briefly, 1 g of soil was extracted sequentially by 0.25 mol·L^-1^ NaHCO_3_ solution, 0.5 mol·L^-1^ NH_4_OAc solution, 0.1 mol·L^-1^NaOH - 0.1 mol·L^-1^ NaCO_3_ solution, a mixed solution of 40 mL 0.3mol·L^-1^ C_6_H_5_Na_3_O_7_ and 10 mL 0.5 mol·L^-1^ NaOH, 0.5 mol·L^-1^ 1/2 H_2_SO_4_ solution and then measured by molybdenum blue colorimetric method to obtain Ca_2_-P content, Ca_8_-P content, Al-P content, Fe-P content, O-P and Ca_10_-P in turn. Organic P was determined by the Scorching method (Bao, 2000). Briefly, both the soil subsample scorched at 550°C (to convert organic P into inorganic P) and the soil subsample without scorching were leached with 0.2 mol·L^-1^ 1/2 H_2_SO_4_ solution, and the inorganic P content was determined. The difference in inorganic P content between the scorched and unscorched subsamples was the organic P content.

### 2.6 Statistical analysis

Statistical analyses were conducted using SPSS 18.0 (SPSS Inc., Chicago, IL, USA) and R v.4.3.1 software (http://cran.r-project.org). All figures were drawn with Origin 2017 software (Origin Lab Inc., Northampton, MA, USA). Normal distribution and chi-square tests were performed on the data before analysis and logarithmic conversion was performed if necessary. Significant differences in dry matter weight (Dw), N accumulation (Na), P accumulation (Pa), K accumulation (Ka), CAT, POD, SOD, MDA, soil C fractions and soil P fractions among treatments were tested by one-way and two-way analysis of variance (ANOVA) followed by Duncan’s multiple range test. The effects of straw return, P addition, soil C and P fractions on plant growth factors (antioxidant enzyme activity, MDA content, nutrient accumulation and Dw) were fitted using structural equation model. The structural equation model was analyzed at Amos 28.0 (SPSS Inc., Chicago, IL, USA). The response ratio (RR) of each variable to straw returning (or P addition) was calculated as the *ln*-ratio of the value in the straw returning (or P addition) treatment to that in the corresponding control. One-sample T test was used to test the significant differences between the RR value and value “0”. Statistical significance was set at *p* < 0.05. All data are presented as the mean ± standard error (SE).

## 3 Results

### 3.1 Dry matter weight and nutrient accumulation of maize

**Figure 1:**
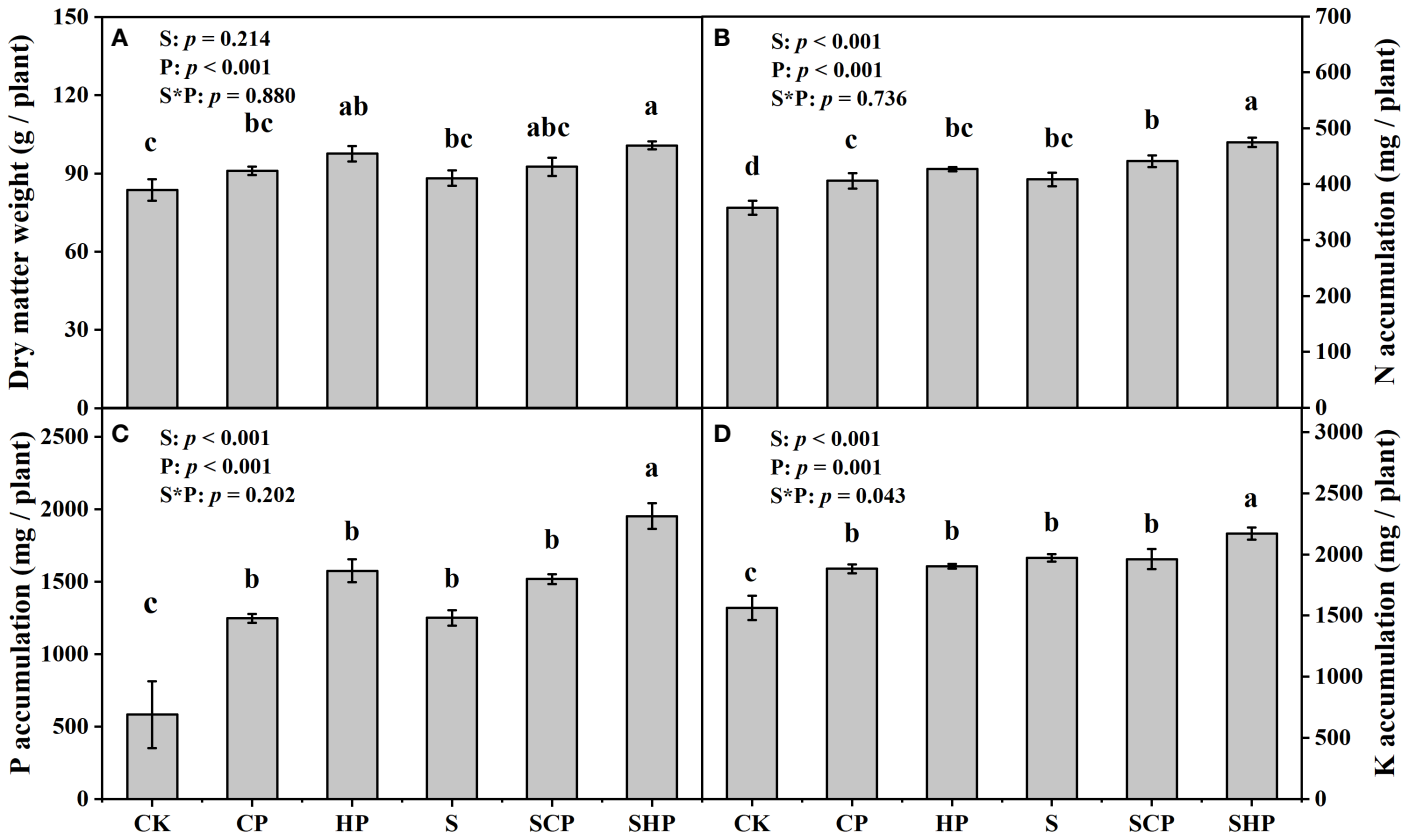
Dry matter weight **(A)** and N **(B)**, P **(C)** and K **(D)** accumulation of maize at the mature stage under different treatments. Error bars indicate standard error (SE, n = 4). Different lowercase letters above the bars denote significant differences among treatments based on one-way ANOVA using Duncan’s multiple range test (*p* < 0.05). The results of two-way ANOVA (straw application levels* P fertilizer levels) are shown in the top left corner of the figures. S, straw application; P, P fertilizer; S*P, the interaction of straw and P applications. CK, without straw or P application; CP, conventional P application without straw return; HP, high P application without straw return; S, straw return without P application; SCP, straw return with conventional P application; SHP, straw return with high P application.

The addition of straw had no significant effect on maize dry matter weight but increased N, P and K accumulations by 11.21%, 38.62% and 14.13%, respectively (*p* < 0.05) (**Figure 1**; **Supplementary Figure 1**). The RRs of nutrient accumulations to straw addition were highest in no P fertilizer (Non-P) level (**Supplementary Figure 1**). Dry matter weight and nutrient accumulations increased with increasing P application rate, but the trends were not consistent across the different straw conditions: the accumulation of N, P and K all showed high application rate of P fertilizer (HP) = conventional application rate of P fertilizer (CP) > Non-P under the condition without straw addition and showed HP > CP = Non-P under the condition with straw addition (*p* < 0.05) (**Figure 1**). Generally, HP soils had higher RRs of dry matter weight and nutrient accumulations than CP soils (**Supplementary Figure 2**).

### 3.2 Antioxidant enzyme activity and MDA content of maize plants

**Figure 2:**
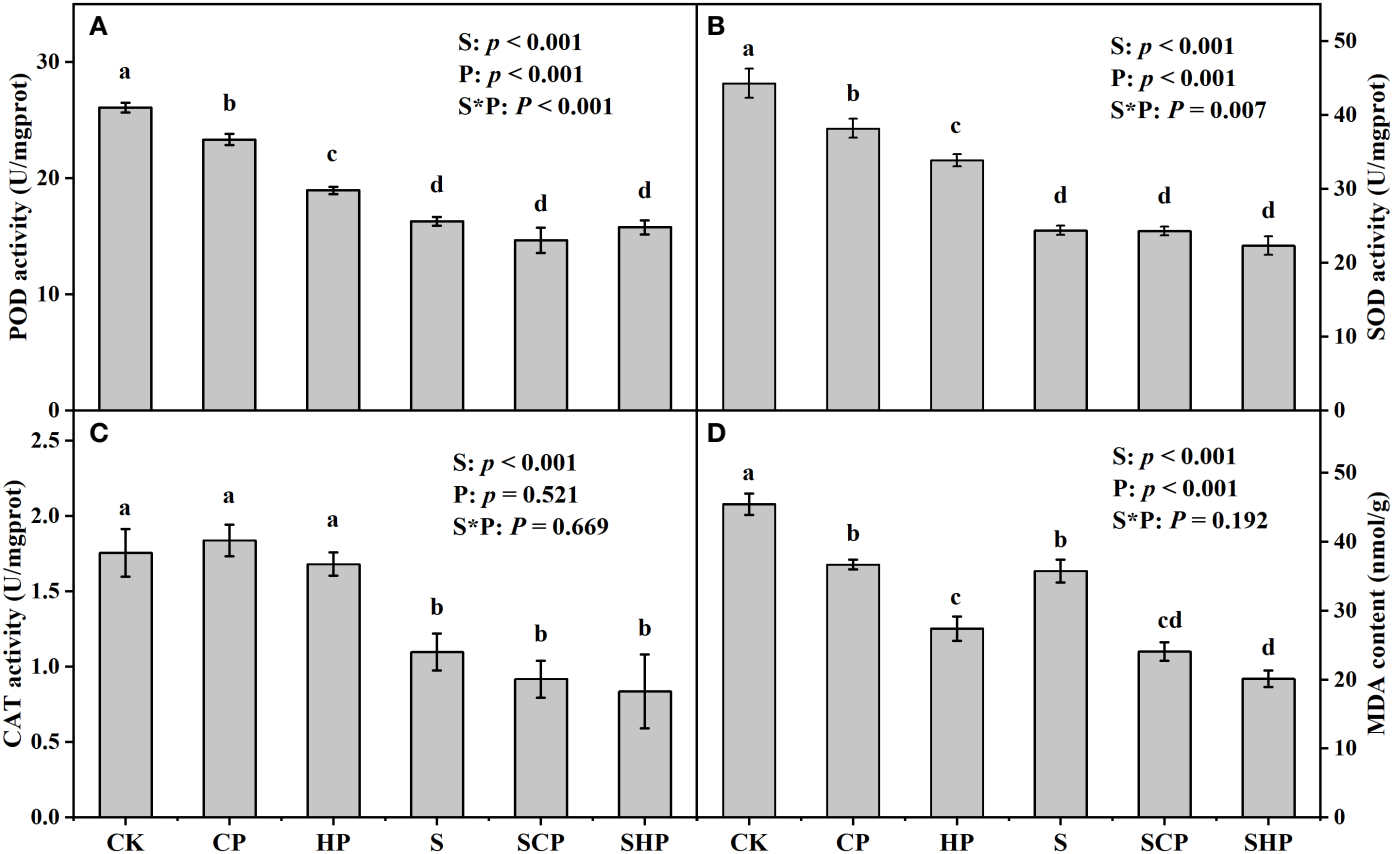
POD **(A)**, SOD **(B)**, CAT **(C)** activity and MDA content **(D)** of maize plants at the tasseling stage under different treatments. Error bars indicate standard error (SE, n = 4). Different lowercase letters above the bars denote significant differences among treatments based on one-way ANOVA using Duncan’s multiple range test (*p* < 0.05). The results of two-way ANOVA (straw application levels* P fertilizer levels) are shown in the top right corner of the figures. POD, peroxidase; SOD, superoxide dismutase; CAT, catalase; MDA, malondialdehyde. For other abbreviations, see **Figure 1**.

Straw application reduced the activities of POD, SOD, CAT and the content of MDA in maize plants by 31.69%, 38.99%, 45.96% and 27.04%, respectively (*p* < 0.05) (**Figure 2**). The RRs of POD, SOD, CAT and MDA to straw return was negative (**Supplementary Figure 1**). SOD activity, POD activity and MDA content decreased with increasing P application rate in the absence of straw. In treatments with straw addition, the MDA content of the S treatment was 48.65% and 77.89% higher than that of the SCP and SHP treatments, respectively (*p* < 0.05) (**Figure 2**). In straw-absent soils, the RRs of POD, SOD and MDA to P addition were much greater under HP level than under CP level (**Supplementary Figure 2A**).

### 3.3 SOC content and fractions

**Figure 3:**
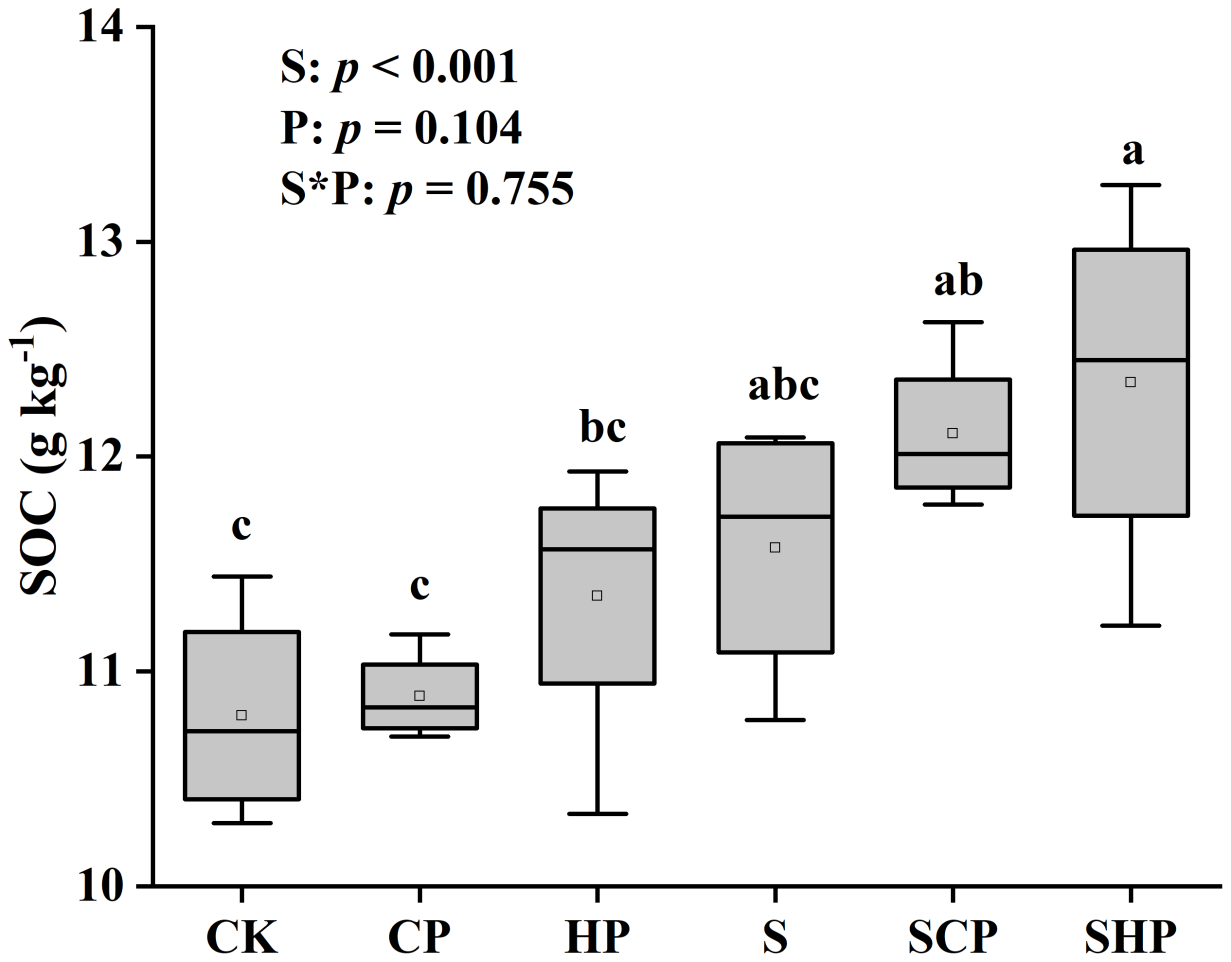
SOC content at the mature stage under different treatments. Within the box plot charts, the crosspieces of each box plot represent (from top to bottom) the maximum, upper-quartile, median (black bar), lower-quartile and minimum values. Different lowercase letters above the bars denote significant differences among treatments based on one-way ANOVA using Duncan’s multiple range test (*p* < 0.05). The results of two-way ANOVA (straw application levels* P fertilizer levels) are shown in the top left corner of the figures. SOC, soil organic carbon. For other abbreviations, see **Figure 1**.

**Figure 4:**
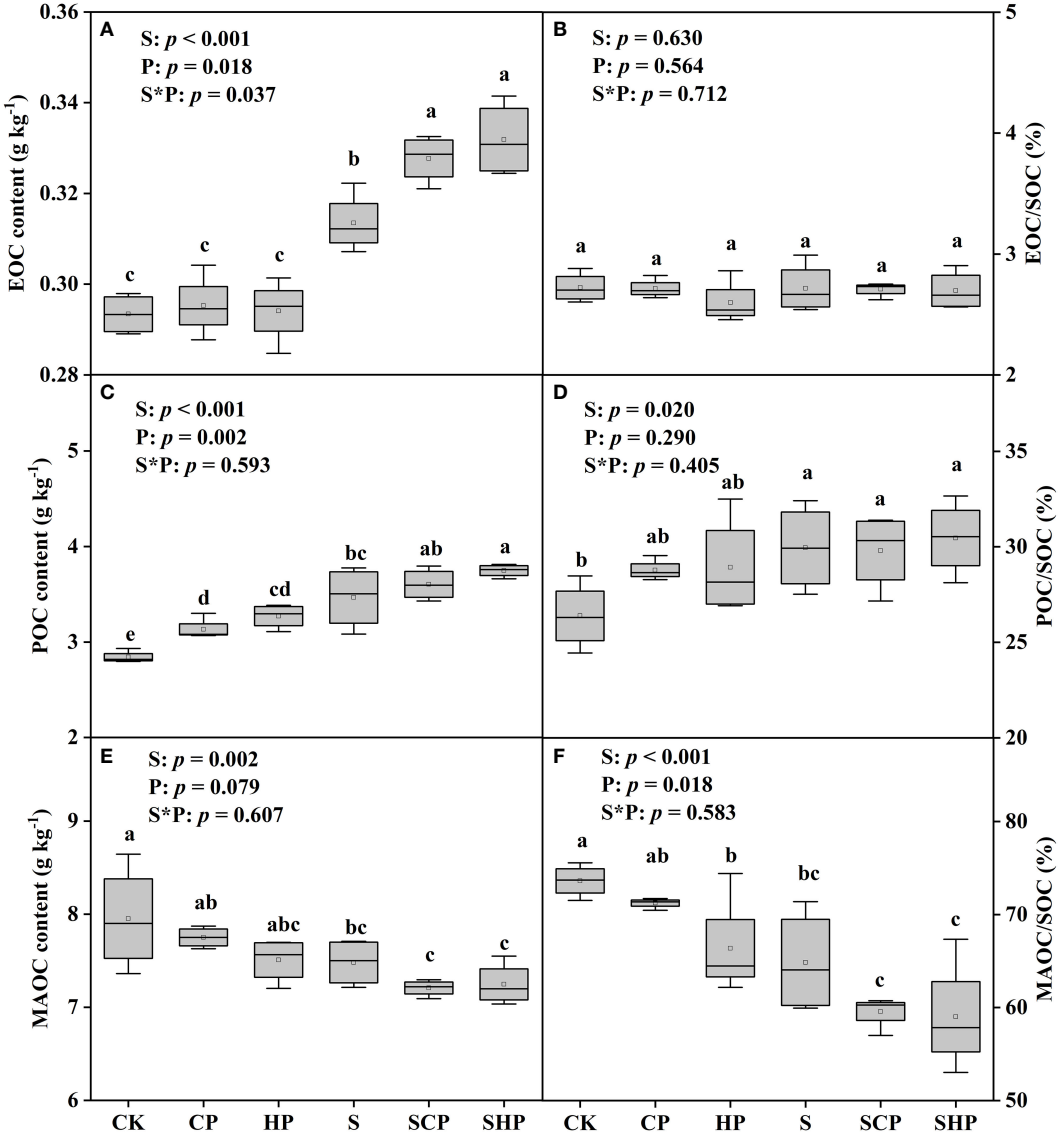
Soil EOC, POC and MAOC contents **(A, C, E)** and their proportions to SOC **(B, D, F)** under different treatments. Within the box plot charts, the crosspieces of each box plot represent (from top to bottom) the maximum, upper-quartile, median (black bar), lower-quartile and minimum values. Different lowercase letters above the bars denote significant differences among treatments based on one-way ANOVA using Duncan’s multiple range test (*p* < 0.05). The results of two-way ANOVA (straw application levels* P fertilizer levels) are shown in the top left corner of the figures. EOC, easily oxidized organic carbon; POC, particulate organic carbon; MAOC, mineral-associated organic carbon; SOC, soil organic carbon. For other abbreviations, see **Figure 1**.

The SOC, EOC, POC contents and POC/SOC ratio in straw-added soils were on average 9.07%, 10.23%, 17.00% and 7.27% higher than those in non-straw soils, while the corresponding MAOC content and MAOC/SOC ratio were much lower (*p* < 0.05) (**Figures 3**, **4**). EOC/SOC ratio had no difference among all treatments (**Figure 4**). The effects of P application rate on soil C fraction were different with C fractions and straw return levels: under the condition without straw addition, there was no significant difference in soil EOC content among the three P application rates, but the POC content of the CP and HP treatments was 10.31% and 15.18% higher than that of the CK treatment, respectively; under the condition of straw addition, the soil EOC and POC contents increased with increasing P application rate, but no significant change in EOC/SOC or POC/SOC was found. Regardless of the straw addition levels, P application tended to decrease soil MAOC content and MAOC/SOC ratio (*p* < 0.05) (**Figure 4**).

### 3.4 Soil phosphorus fractions

**Figure 5:**
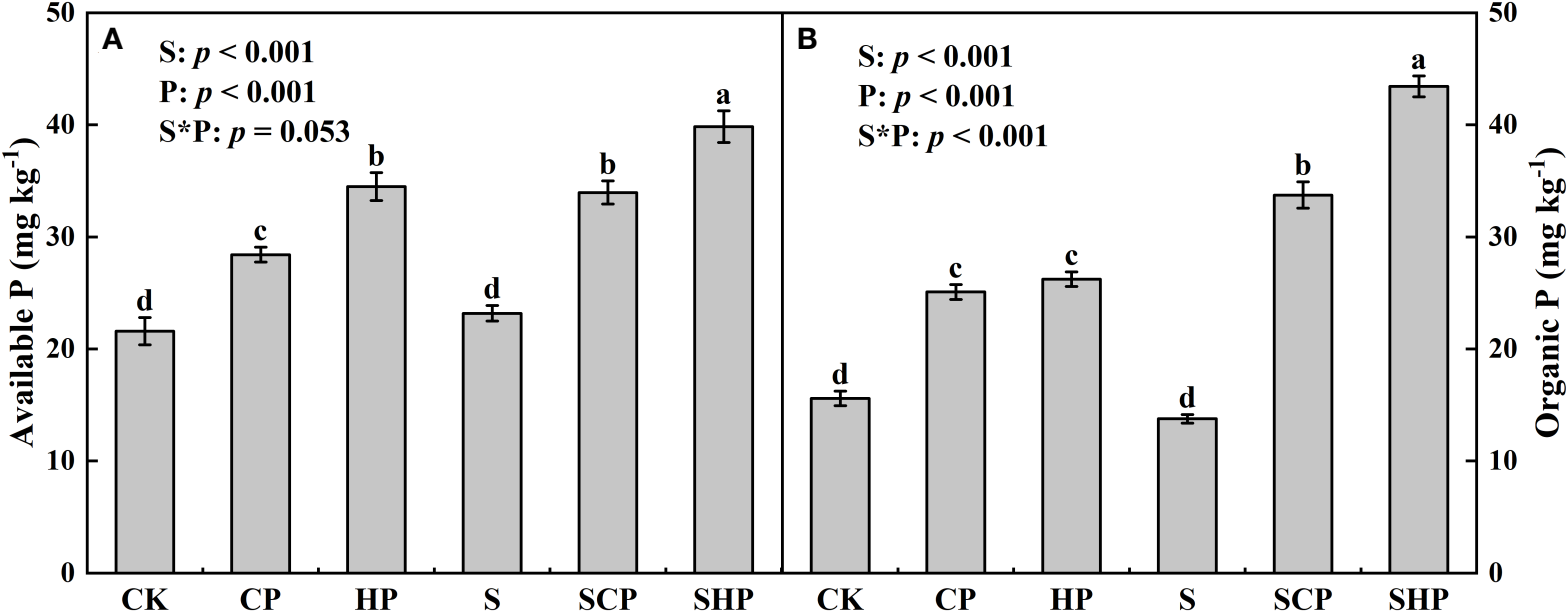
Soil available P **(A)** and organic P **(B)** contents at the mature stage of maize under different treatments. Error bars indicate standard error (SE, n = 4). Different lowercase letters above the bars denote significant differences among treatments based on one-way ANOVA using Duncan’s multiple range test (*p* < 0.05). The results of two-way ANOVA (straw application levels* P fertilizer levels) are shown in the top left corner of the figures. For abbreviations, see **Figure 1**.

The effects of straw return on soil P fractions varied with P application rates and P fraction types. For soils without P addition, the contents of AP, organic P and total inorganic P (TIP) were not affected by straw return, whereas positive effects were found in P-added soils. The soil AP content of the SCP and SHP treatments was 19.52% and 15.52% higher than that of the CP and HP treatments, respectively, and the corresponding increases in the soil organic P content were 34.44% and 65.59%, respectively. Straw return raised the soil TIP content by 3.63% and 3.60% at the CP and HP application rates, respectively (*p* < 0.05) (**Figure 5**; **Supplementary Table 1**). Except for Ca_2_-P, the proportions of inorganic P fractions to TIP were not affected by straw return (*p* > 0.05) (**Supplementary Table 1**). With the increase in the P application rate, the proportions of Ca_2_-P and O-P to TIP increased, while the proportion of Ca_10_-P decreased.

### 3.5 Factors affecting maize dry matter weight

**Figure 6:**
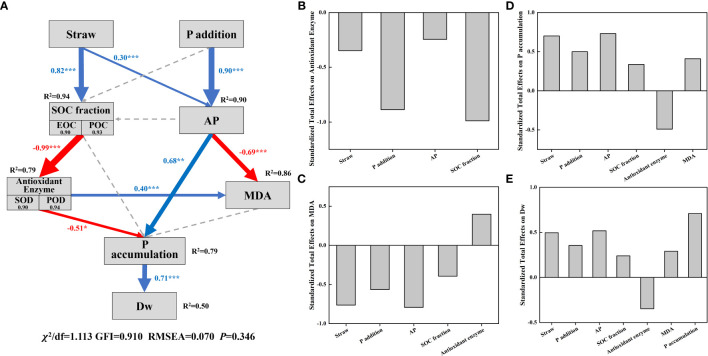
A structural equation model **(A)** fitted to plant growth conditions, soil C fractions and soil P fractions. The standardized total effect of different factors on antioxidant enzyme **(B)**, MDA content **(C)**, P accumulation **(D)** and plant dry matter weigh **(E)**. The number in the box represents the Spearman correlation coefficient between this variable and the composite variable after PCA. The thickness of the arrow represents the significance of the relationship, the blue arrow represents the positive correlation, the red arrow represents the negative correlation, and gray dashed arrows represent non-significant paths. *p < 0.05; **p < 0.01; ***p < 0.001. The numbers in the arrows are normalized path coefficients. R^2^ represents the degree of explanation for the variability of the response variable. SOC, soil organic carbon; EOC, easily oxidized organic carbon; POC, particulate organic carbon; AP, soil available P; POD, peroxidase; SOD, superoxide dismutase; MDA, malondialdehyde; Dw, dry matter weight.

A structural equation model (SEM) was conducted to fit the relationships among plant growth factors, soil C and P fractions (**Figure 6**). The results showed that straw return, P addition, AP, and soil C fractions showed negative effects on MDA, with AP showing the greatest negative effect on that. These variables, together with antioxidant enzyme, explain 86% of the total variance in MDA. Straw return, P addition, AP, soil C fractions, MDA and P accumulation showed positive effects, while antioxidant enzymes showed negative effects on Dw. Particularly, P accumulation had the largest positive effect on Dw. Generally, straw return and P addition altered antioxidant enzyme levels, MDA content, nutrient accumulation and dry matter weight of maize plants through changing soil C fractions (EOC and POC) and P availability.

## 4 Discussion

### 4.1 Effect of straw and P applications on dry matter weight and nutrient accumulation of maize

Our results showed that straw addition had no significant effect on the dry matter weight of maize but significantly increased N, P and K accumulations (*p* < 0.05) (**Figure 1**; **Supplementary Figure 1**), indicating that straw return can effectively improve nutrient use efficiencies. This may be because straw return provides more substrates for microbes and stimulates microbial activity, which accelerates the process of nutrient mineralization by microorganisms and increases the nutrient availability of the soil, which in turn increases the nutrient accumulation of maize. It was confirmed by the finding of Cao et al. (2021) that straw addition increased the contents of available N (AN), available P (AP) and available K (AK) in saline soils. Our results showed that straw return is an important measure to improve crop growth and nutrient uptake in saline soil.

In this study, a high application rate of P (HP) increased the N, P and K accumulations and dry matter weight of maize regardless of straw return, while the conventional application rate of P (CP) only increased the N, P and K accumulations of maize when straw was not applied (**Figure 1**; **Supplementary Figure 2**). Considering the high content of CaCO_3_ in saline soils of the Yellow River Delta, which strongly fixes water-soluble P and reduces soil P availability (Mahmood et al., 2013), it is reasonable to obtain the result that P addition (regardless of the conventional or high application rate) promotes maize growth and nutrient accumulation. However, the promoting effects of conventional P application on plant nutrient accumulation might be covered by the straw return management, because straw return not only bring plant-derived P into soil but also active soil original P.

### 4.2 Effect of straw and P applications on antioxidant enzyme activity and MDA content of maize

It is well known that SOD, CAT, POD and MDA are all major indicators of crop stress tolerance, in which SOD, CAT and POD can breakdown the peroxides and superoxide anions produced in plants and mitigate the physiological damage caused by environmental stresses, and MDA (one of the products of lipid peroxidation) can reflect the extent of oxidative damage to the cell membrane (Miller et al., 2010). Our data showed that straw return significantly reduced SOD, CAT, POD activities and MDA content in maize leaves (*p* < 0.05) (**Figure 2**; **Supplementary Figure 1**). This may be because straw addition increases soil cation exchange capacity (CEC) and ion adsorption capacity, thereby reducing the soluble salt content of the soil and decreasing the salt stress to maize (Leogrande and Vitti, 2019). Previous study by Khan et al. (2019) found that straw application in an Orthic Anthorsols reduced the MDA content of the crop but induced an increase in both SOD and POD activities. We speculate that this inconsistency is related to the fact that the plants experienced environmental stress in our study but not in their study (Khan et al., 2019). Furthermore, the organic acids produced during straw decomposition could promote the production of soil exchangeable Ca^2+^, which is beneficial for increasing cell membrane stability. Therefore, the plant is less physiologically damaged, and the antioxidant enzyme activity is reduced after straw return.

The POD and SOD activities of maize decreased with increasing P application rates in the absence of straw; however, no significant difference in antioxidant enzyme activities (i.e., SOD, POD, CAT) was found among the different P fertilizer levels after straw addition (*p* > 0.05) (**Figure 2**; **Supplementary Figure 2**). This may be because straw return reduces the salt stress to the crop and downregulates the activity of antioxidant enzymes, as mentioned above; thus, the antioxidant levels of the crop were not sensitive to P application. The MDA content decreased with increasing P application rate in our study (**Figure 2D**), confirming the stabilizing effect of P fertilizer on cell membrane structure (Zhong et al., 2018).

### 4.3 Effect of straw and P applications on SOC fractions

Our results showed that straw addition increased the contents of SOC, EOC and POC, and decreased the content of MAOC (**Figures 3**, **4**). Wu et al. (2021) also found that straw return can enhance the unstable C fraction (i.e., POC, EOC) of soil. This may be attributed to the fact that the labile C fraction is more sensitive to field management practices and the high level of polysaccharides (cellulose and hemicellulose) in straw (Ghosh et al., 2012). The effect of straw return on MAOC content has great variability. For instance, He et al. (2022) found that straw return could increase MAOC content in low-salinity soil and medium-salinity soil, whereas He et al. (2015) found that straw return may have either increased, decreased, or no significant effect on MAOC content under three different climates. It was found that straw addition increased the proportion of POC in SOC, while decreased that of MAOC in SOC (**Figure 4**). The decrease in MAOC content and MAOC/SOC may be due to the positive priming effect of SOC mineralization caused by straw addition, as indicated by the finding of Dai et al. (2022) that the positive priming effect was accompanied by a decrease in MAOC content. Straw return might lead to more MAOC decomposition rather than synthesis in saline soils, partly because excess Na^+^ can dislodge organic matter from clay particles.

We found that P application (particularly at a high rate) led to an increase in EOC and POC content (**Figure 4**). This may be attributed to the following reasons: i) P addition stimulates soil microbial activity (Rooney and Clipson, 2009) and increases the synthesis of extracellular enzymes (Dong et al., 2015), and ii) P application promotes desorption of organic C adsorbed on soil colloids and alleviates microbial C limitation (Spohn and Schleuss, 2019), thus accelerating the turnover of soil C pools and resulting in more active and less stable C fractions (Sun et al., 2017). Our results showed that P application decreased the contribution of MAOC to the SOC pool (**Figure 4**). Similar to our findings, Yuan et al. (2021) found that P addition decreases the contribution of microbial residuals, which are considered to be the predominant source of MAOC (Samson et al., 2020), to the SOC pool, indicating that P application may reduce SOC stability.

### 4.4 Effect of straw application on soil P availability

Straw addition generally increased soil P availability in our study, as indicated by the increases in soil available P, organic P and Ca_2_-P contents (*p* < 0.05) (**Figure 5**; **Supplementary Figure 1**; **Supplementary Table 1**), showing a strong ability to activate soil P. Soil P availability depends mainly on the dissolution of stationary P (i.e. insoluble P compounds in the soil and P held in clay particles) and the mineralization of organic P (Jing et al., 2017). Straw return can significantly improve soil hydrothermal conditions (i.e., soil moisture and soil temperature) (Wang et al., 2019), increase organic acid exudation (Sun et al., 2020), and effectively enhance microbial proliferation and promote phosphatase production (Guan et al., 2020), thus directly or indirectly affecting the soil P activation process. Our results are consistent with the findings of Cao et al. (2021); Li et al. (2015) in saline soil and andosol, respectively.

### 4.5 Factors affecting the growth of maize plants

The SEM results showed that SOC fractions (including EOC and POC) had a high negative standardized total effect on antioxidant enzyme (including SOD and POD) activities of plants, and soil AP was negatively correlated with plant MDA content (**Figure 6**), suggesting an important role of SOC fractions and P availability in plant resistance to salt stress. In addition, AP had the highest and positively standardized total effect on plant P accumulation (**Figure 6**). These results reflected the improvement effect of P on plant growth. Saline soil in the Yellow River Delta is characterized by severe P deficiency and low biological productivity. The addition of straw and P fertilizer increased soil AP content, thereby improving the absorption of P by maize and promoting plant growth (Chen et al., 2021). Our results echoed the findings of Ding et al. (2020) that the combined application of P and organic amendments could reduce the negative impact of salt on wheat and increase the accumulation of P, thus improving the yield and quality of wheat.

## 5 Conclusion

**Figure 7:**
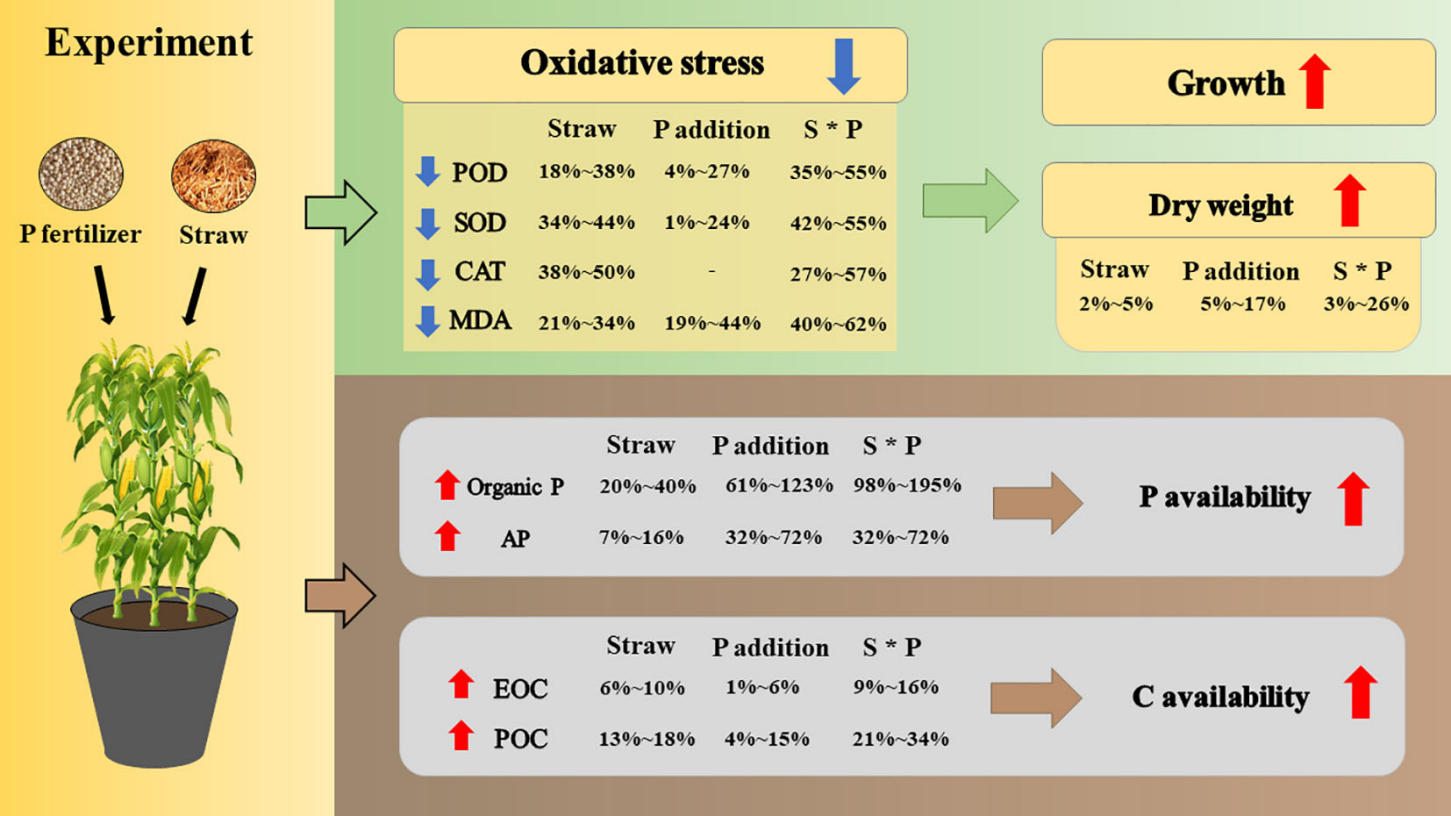
Conceptual diagram of the impact of straw return and P fertilization on plant properties and soil properties. In the concept diagram, red solid arrows indicate an increase and blue solid arrows indicate a decrease. “-” represents that P addition has no uniform positive/negative effect on CAT activity. For abbreviations, see **Figure 6**.

In this study, straw return with P application (particularly at a high rate) improved the nutrient accumulations of maize and alleviated the oxidative damage of maize plants from salt stress. Both straw return and P supply increased the POC content of saline soils while decreased the contribution of MAOC to SOC, indicating the acceleration of SOC turnover. Meanwhile, straw return improved soil P availability, as suggested by the increases in the contents of soil available P and organic P. The reduction effects of P application on plant antioxidant enzyme activities were buffered, while the increasing effect of that on soil P availability was enhanced by straw return. These results suggest that straw return together with adequate P supply could play important roles in alleviating oxidation damage on plants and activating the C and P pools of saline soils (**Figure 7**). Future studies should pay more attention to the microbial participation mechanisms in these processes for a better understanding of soil C-P interactions, and on the other hand, long-term open field experiment should be conducted to test the generality of these findings.

## Data availability statement

The original contributions presented in the study are included in the article/**Supplementary Material**. Further inquiries can be directed to the corresponding authors.

## Author contributions

ZG: Data curation, Formal analysis, Investigation, Writing – original draft. WY: Data curation, Formal analysis, Investigation, Writing – original draft. HW: Conceptualization, Funding acquisition, Project administration, Writing – review & editing. WH: Formal analysis, Writing – original draft. YT: Visualization, Writing – original draft. GH: Resources, Writing – review & editing. YL: Methodology, Writing – review & editing. HP: Software, Writing – review & editing. QY: Software, Writing – review & editing. YZ: Conceptualization, Funding acquisition, Project administration, Writing – review & editing.

## References

[B1] Adams J. L. Tipping E. Bryant C. L. Helliwell R. C. Toberman H. Quinton J. (2015). Aged riverine particulate organic carbon in four UK catchments. Sci. Total. Environ. 536, 648–654. doi: 10.1016/j.scitotenv.2015.06.141 26254066

[B2] Bao S. D. (2000). “Determination of phosphorus in soil” and “Determination of plant ash and various nutrient elements”, Soil analysis in agricultural chemistry (Beijing: China Agriculture Press).

[B3] Bu R. Ren T. Lei M. Liu B. Li X. Cong R.. (2020). Tillage and straw-returning practices effect on soil dissolved organic matter, aggregate fraction and bacteria community under rice-rice-rapeseed rotation system. Agric. Ecosyst. Environ. 287, 106681. doi: 10.1016/j.agee.2019.106681

[B4] Cai A. Feng W. Zhang W. Xu M. (2016). Climate, soil texture, and soil types affect the contributions of fine-fraction-stabilized carbon to total soil organic carbon in different land uses across China. J. Environ. Manage. 172, 2–9. doi: 10.1016/j.jenvman.2016.02.009 26905446

[B5] Cao N. Wang J. Pang J. Hu W. Bai H. Zhou Z.. (2021). Straw retention coupled with mineral phosphorus fertilizer for reducing phosphorus fertilizer input and improving cotton yield in coastal saline soils. Field. Crop Res. 274, 108309. doi: 10.1016/j.fcr.2021.108309

[B6] Chen M. Zhang S. Liu L. Wu L. Ding X. (2021). Combined organic amendments and mineral fertilizer application increase rice yield by improving soil structure, P availability and root growth in saline-alkaline soil. Soil Tillage Res. 212, 105060. doi: 10.1016/j.still.2021.105060

[B7] Dai S. He P. Guo X. Ge T. Oliver M. A. Li L. (2022). Faster carbon turnover in topsoil with straw addition is less beneficial to carbon sequestration than subsoil and mixed soil. Soil. Sci. Soc Am. J. 86, 1431–1443. doi: 10.1002/saj2.20412

[B8] Damon P. M. Bowden B. Rose T. Rengel Z. (2014). Crop residue contributions to phosphorus pools in agricultural soils: A review. Soil. Biol. Biochem. 74, 127–137. doi: 10.1016/j.soilbio.2014.03.003

[B9] Ding Z. Kheir A. M. S. Ali M. G. M. Ali O. A. M. Abdelaal A. I. N. Lin X. E.. (2020). The integrated effect of salinity, organic amendments, phosphorus fertilizers, and deficit irrigation on soil properties, phosphorus fractionation and wheat productivity. Sci. Rep. 10, 2736. doi: 10.1038/s41598-020-59650-8 32066858 PMC7026061

[B10] Dong W. Zhang X. Liu X. Fu X. Chen F. Wang H.. (2015). Responses of soil microbial communities and enzyme activities to nitrogen and phosphorus additions in Chinese fir plantations of subtropical China. Biogeosciences. 12, 5537–5546. doi: 10.5194/bg-12-5537-2015

[B11] Food and Agriculture Organization of the United Nations (2021) The state of the world’s land and water resources for food and agriculture – Systems at breaking point. Synthesis report 2021 (Rome) (Accessed December 11, 2021).

[B12] Ghosh S. Wilson B. Ghoshal S. Senapati N. Mandal B. (2012). Organic amendments influence soil quality and carbon sequestration in the Indo-Gangetic plains of India. Agric. Ecosyst. Environ. 156, 134–141. doi: 10.1016/j.agee.2012.05.009

[B13] Gu Y. Jiang B. (1990). Determination of inorganic phosphorus classification of limy soils. Soil 2, 101–102.

[B14] Guan X. Wei L. Turner N. C. Ma S. Yang M. Wang T. (2020). Improved straw management practices promote *in situ* straw decomposition and nutrient release, and increase crop production. J. Clean. Prod. 250, 119514. doi: 10.1016/j.jclepro.2019.119514

[B15] Guo D. Huang Z. Zhang K. Zhang S. Song X. Wang B.. (2018). Effects of long-term synergistic use of organic and inorganic exogenous P on phosphorus availability in fluvo-aquic soil. J. Of. Plant Nutr. And. Fertilizers. 24, 1651–1659. doi: 10.11674/zwyf.18226

[B16] He W. Wang H. Ye W. Tian Y. Hu G. Lou Y.. (2022). Distinct stabilization characteristics of organic carbon in coastal salt-affected soils with different salinity under straw return management. Land. Degrad. Dev. 33, 2246–2257. doi: 10.1002/ldr.4276

[B17] He Y. Zhang W. Xu M. Tong X. Sun F. Wang J.. (2015). Long-term combined chemical and manure fertilizations increase soil organic carbon and total nitrogen in aggregate fractions at three typical cropland soils in China. Sci. Total. Environ. 532, 635–644. doi: 10.1016/j.scitotenv.2015.06.011 26119378

[B18] Jing Z. Chen R. Wei S. Feng Y. Zhang J. Lin X. (2017). Response and feedback of C mineralization to P availability driven by soil microorganisms. Soil. Biol. Biochem. 105, 111–120. doi: 10.1016/j.soilbio.2016.11.014

[B19] Khan M. N. Lan Z. Sial T. A. Zhao Y. Haseeb A. Jianguo Z.. (2019). Straw and biochar effects on soil properties and tomato seedling growth under different moisture levels. Arch. Agron. Soil. Sci. 65, 1704–1719. doi: 10.1080/03650340.2019.1575510

[B20] Kirkby C. A. Richardson A. E. Wade L. J. Passioura J. B. Batten G. D. Blanchard C.. (2014). Nutrient availability limits carbon sequestration in arable soils. Soil. Biol. Biochem. 68, 402–409. doi: 10.1016/j.soilbio.2013.09.032

[B21] Lakhdar A. Hafsi C. Rabhi M. Debez A. Montemurro F. Abdelly C.. (2008). Application of municipal solid waste compost reduces the negative effects of saline water in Hordeum maritimum L. Bioresource. Technol. 99, 7160–7167. doi: 10.1016/j.biortech.2007.12.071 18308562

[B22] Leogrande R. Vitti C. (2019). Use of organic amendments to reclaim saline and sodic soils: a review. Arid. Land. Res. Manage. 33, 1–21. doi: 10.1080/15324982.2018.1498038

[B23] Li K. Li Q. Geng Y. Liu C. (2020). An evaluation of the effects of microstructural characteristics and frost heave on the remediation of saline-alkali soils in the Yellow River Delta, China. Land Degrad. Dev. 32, 1325–1337. doi: 10.1002/ldr.3801

[B24] Li X. Li F. Singh B. Cui Z. Rengel Z. (2006). Decomposition of maize straw in saline soil. Biol. Fert. Soils. 42, 366–370. doi: 10.1007/s00374-005-0042-9

[B25] Li S. Li X. Zhu W. Chen J. Tian X. Shi J. (2019). Does straw return strategy influence soil carbon sequestration and labile fractions? Agron. J. 111, 897–906. doi: 10.2134/agronj2018.08.0484

[B26] Li Y. Yang R. Gao R. Wei H. Chen A. Li Y. (2015). Effects of long-term phosphorus fertilization and straw incorporation on phosphorus fractions in subtropical paddy soil. J. Integr. Agric. 14, 365–373. doi: 10.1016/S2095-3119(13)60684-X

[B27] Mahmood I. Ali A. Gill M. Shahzad A. Sultan T. Hussain F. (2013). Phosphorus availability in different salt-affected soils as influenced by crop residue incorporation. Int. J. Agric. Biol. 15, 472–478.

[B28] Miller G. Suzuki N. Ciftci-Yilmaz S. Mittler R. (2010). Reactive oxygen species homeostasis and signalling during drought and salinity stresses. Plant Cell. Environ. 33, 453–467. doi: 10.1111/j.1365-3040.2009.02041.x 19712065

[B29] Ouni Y. Lakhdar A. Scelza R. Scotti R. Abdelly C. Barhoumi Z.. (2013). Effects of two composts and two grasses on microbial biomass and biological activity in a salt-affected soil. Ecol. Eng. 60, 363–369. doi: 10.1016/j.ecoleng.2013.09.002

[B30] Pikul J. L. Osborne S. Ellsbury M. Riedell W. (2007). Particulate organic matter and water-stable aggregation of soil under contrasting management. Soil. Sci. Soc Am. J. 71, 766–776. doi: 10.2136/sssaj2005.0334

[B31] Qu X. Wang X. Wu J. He P. (2021). Both carbon sequestration and yield are related to particulate organic carbon stability affected by organic amendment origins in mollisol. J. Soils. Sediments. 21, 3044–3056. doi: 10.1007/s11368-021-03010-0

[B32] Rooney D. C. Clipson N. J. W. (2009). Phosphate addition and plant species alters microbial community structure in acidic upland grassland soil. Microb. Ecol. 57, 4–13. doi: 10.1007/s00248-008-9399-2 18581037

[B33] Samson M. Chantigny M. H. Vanasse A. Menasseri-Aubry S. Royer I. Angers D. A. (2020). Management practices differently affect particulate and mineral-associated organic matter and their precursors in arable soils. Soil. Biol. Biochem. 148, 107867. doi: 10.1016/j.soilbio.2020.107867

[B34] Setia R. Gottschalk P. Smith P. Marschner P. Baldock J. Setia D.. (2013). Soil salinity decreases global soil organic carbon stocks. Sci. Total. Environ. 465, 267–272. doi: 10.1016/j.scitotenv.2012.08.028 22959898

[B35] Spohn M. Schleuss P. (2019). Addition of inorganic phosphorus to soil leads to desorption of organic compounds and thus to increased soil respiration. Soil. Biol. Biochem. 130, 220–226. doi: 10.1016/j.soilbio.2018.12.018

[B36] Sun X. Shen J. Wang Z. Liu M. Lou L. Yue C.. (2017). Effect of exogenous phosphorus input on the availability and turnover characteristics of soil carbon pool in agro-riparian wetlands. Chinese. J. Of. Eco-Agriculture. 25, 1433–1443. doi: 10.13930/j.cnki.cjea.170266

[B37] Sun C. Wang D. Shen X. Li C. Liu J. Lan T.. (2020). Effects of biochar, compost and straw input on root exudation of maize (*Zea mays* L.): From function to morphology. Agric. Ecosyst. Environ. 297, 106952. doi: 10.1016/j.agee.2020.106952

[B38] Wang W. Akhtar K. Ren G. Yang G. Feng Y. Yuan L. (2019). Impact of straw management on seasonal soil carbon dioxide emissions, soil water content, and temperature in a semi-arid region of China. Sci. Total. Environ. 652, 471–482. doi: 10.1016/j.scitotenv.2018.10.207 30368177

[B39] Wang X. Qi P. Cai L. Chen X. Xie J. Gan H.. (2020). Effects of alternative fertilization practices on components of the soil organic carbon pool and yield stability in rain-fed maize production on the Loess Plateau. Acta Prataculturae. Sinica. 29, 58–69. doi: 10.11686/cyxb2020187

[B40] Weil R. R. Islam K. R. Stine M. A. Gruver J. B. Samson-Liebig S. E. (2003). Estimating active carbon for soil quality assessment: A simplified method for laboratory and field use. Am. J. Alternative. Agr. 18, 3–17. doi: 10.1079/AJAA200228

[B41] Wu L. Zhang S. Ma R. Chen M. Wei W. Ding X. (2021). Carbon sequestration under different organic amendments in saline-alkaline soils. Catena. 196, 104882. doi: 10.1016/j.catena.2020.104882

[B42] Xie W. Wu L. Zhang Y. Wu T. Li X. Ouyang Z. (2017). Effects of straw application on coastal saline topsoil salinity and wheat yield trend. Soil. Till. Res. 169, 1–6. doi: 10.1016/j.still.2017.01.007

[B43] Xie W. Zhang Y. Li J. Wei S. Li X. Yu H.. (2021). Straw application coupled with N and P supply enhanced microbial biomass, enzymatic activity, and carbon use efficiency in saline soil. Appl. Soil. Ecol. 168, 104128. doi: 10.1016/j.apsoil.2021.104128

[B44] Xiu L. Zhang W. Sun Y. Wu D. Meng J. Chen W. (2019). Effects of biochar and straw returning on the key cultivation limitations of Albic soil and soybean growth over 2 years. Catena. 173, 481–493. doi: 10.1016/j.catena.2018.10.041

[B45] Yuan Y. Li Y. Mou Z. Kuang L. Wu W. Zhang J.. (2021). Phosphorus addition decreases microbial residual contribution to soil organic carbon pool in a tropical coastal forest. Glob. Change. Biol. 27, 454–466. doi: 10.1111/gcb.15407 33068453

[B46] Zhang F. Chen X. Yao S. Ye Y. Zhang B. (2022a). Responses of soil mineral-associated and particulate organic carbon to carbon input: A meta-analysis. Sci. Total. Environ. 829, 154626. doi: 10.1016/j.scitotenv.2022.154626 35306064

[B47] Zhang M. Li S. Wu X. Zheng F. Song X. Lu J.. (2022b). Nitrogen addition mediates the effect of soil microbial diversity on microbial carbon use efficiency under long-term tillage practices. Land. Degrad. Dev. 33, 2258–2275. doi: 10.1002/ldr.4279

[B48] Zhao S. Yu F. Zhai C. Zhong R. Zhao Y. Wang Y.. (2022). Long-term effects of cattle manure application on the soil aggregate stability of salt-affected soil on the Songnen Plain of North-Eastern China. J. Soils. Sediments 23, 344–354. doi: 10.1007/s11368-022-03317-6

[B49] Zhong G. Wu Z. Liu N. Yin J. (2018). Phosphate alleviation of glyphosate-induced toxicity in *Hydrocharis dubia* (Bl.) Backer. Aquat. Toxicol. 201, 91–98. doi: 10.1016/j.aquatox.2018.05.025 29894895

