# Supplementary material for: Straw and phosphorus applications promote maize (Zea mays L.) growth in saline soil through changing soil carbon and phosphorus fractions
Source: Front Plant Sci. 2024 Jan 19;15:1336300. doi: 10.3389/fpls.2024.1336300 (PMC10835382; doi:10.3389/fpls.2024.1336300)
Supplement: Supplementary file 1 [file DataSheet_1.docx]

**Table S1** The contents of soil total inorganic P and the proportions of soil inorganic P fractions under different treatments.

| Treatments | Total inorganic P | Ca_2_-P proportion | Ca_8_-P proportion | Ca_10_-P proportion | Al-P proportion | Fe-P proportion | O-P proportion |
| --- | --- | --- | --- | --- | --- | --- | --- |
|  | mg kg^-1^ | % | % | % | % | % | % |
| CK | 803.09±4.27d | 1.95±0.08d | 15.32±0.10c | 56.44±0.38a | 7.58±0.18a | 7.67±0.10c | 11.03±0.39a |
| CP | 844.36±3.63c | 2.21±0.05c | 15.87±0.15ab | 54.39±0.15a | 7.45±0.11a | 8.00±0.20b | 12.07±0.16b |
| HP | 889.08±9.72b | 2.80±0.04a | 16.40±0.15a | 52.16±0.27a | 7.40±0.24a | 7.81±0.13a | 13.43±0.27c |
| S | 820.37±6.82d | 1.95±0.03d | 15.60±0.07bc | 56.28±0.19a | 7.47±0.19a | 7.58±0.16c | 11.12±0.40a |
| SCP | 875.00±5.69b | 2.41±0.09b | 15.53±0.16bc | 54.54±0.23a | 7.20±0.17a | 7.80±0.21b | 12.51±0.31b |
| SHP | 921.11±8.41a | 2.71±0.04a | 16.05±0.29ab | 52.14±0.26a | 7.48±0.21a | 7.91±0.12a | 13.72±0.48c |

Note: Different lowercase letters in the same column denote significant differences among treatments according to Duncan's multiple range test (*p* < 0.05). The results are the mean values ± SEs (n = 4). CK, without straw or P application; CP, conventional P application without straw return; HP, high P application without straw return; S, straw return without P application; SCP, straw return with conventional P application; SHP, straw return with high P application.





**Figure S1** Response ratios (RRs) of Dw, Na, Pa, Ka, POD, SOD, CAT, MDA, SOC, EOC, POC, MAOC, AP, OP to straw return at the P0, P1 and P2 levels. Error bars are standard errors (n = 4). Dw, dry matter weight; Na, N accumulation; Pa, P accumulation; Ka, K accumulation; POD, peroxidase; SOD, superoxide dismutase; CAT, catalase; MDA, malondialdehyde; EOC, easily oxidized organic carbon; POC, particulate organic carbon; MAOC, mineral-associated organic carbon; SOC, soil organic carbon; AP, soil available P; OP, soil organic P; Non-P, without P application; CP, conventional P application; HP, high P application.





**Figure S2** Response ratios (RRs) of Dw, Na, Pa, Ka, POD, SOD, CAT, MDA, SOC, EOC, POC, MAOC, AP, OP to P addition (including P1 and P2) in non-straw return (A) and straw return (B) conditions. Error bars are standard errors (n = 4). For abbreviations, see Figure S1.
